# Changes in Gene Expression and Cellular Architecture in an Ovarian Cancer Progression Model

**DOI:** 10.1371/journal.pone.0017676

**Published:** 2011-03-03

**Authors:** Amy L. Creekmore, William T. Silkworth, Daniela Cimini, Roderick V. Jensen, Paul C. Roberts, Eva M. Schmelz

**Affiliations:** 1 Department of Human Nutrition, Foods and Exercise, Virginia Polytechnic Institute and State University, Blacksburg, Virginia, United States of America; 2 Department of Biological Sciences, Virginia Polytechnic Institute and State University, Blacksburg, Virginia, United States of America; 3 Department of Biomedical Science and Pathobiology, Virginia Polytechnic Institute and State University, Blacksburg, Virginia, United States of America; Sanford-Burnham Medical Research Institute, United States of America

## Abstract

**Background:**

Ovarian cancer is the fifth leading cause of cancer deaths among women. Early stage disease often remains undetected due the lack of symptoms and reliable biomarkers. The identification of early genetic changes could provide insights into novel signaling pathways that may be exploited for early detection and treatment.

**Methodology/Principal Findings:**

Mouse ovarian surface epithelial (MOSE) cells were used to identify stage-dependent changes in gene expression levels and signal transduction pathways by mouse whole genome microarray analyses and gene ontology. These cells have undergone spontaneous transformation in cell culture and transitioned from non-tumorigenic to intermediate and aggressive, malignant phenotypes. Significantly changed genes were overrepresented in a number of pathways, most notably the cytoskeleton functional category. Concurrent with gene expression changes, the cytoskeletal architecture became progressively disorganized, resulting in aberrant expression or subcellular distribution of key cytoskeletal regulatory proteins (focal adhesion kinase, α-actinin, and vinculin). The cytoskeletal disorganization was accompanied by altered patterns of serine and tyrosine phosphorylation as well as changed expression and subcellular localization of integral signaling intermediates APC and PKCβII.

**Conclusions/Significance:**

Our studies have identified genes that are aberrantly expressed during MOSE cell neoplastic progression. We show that early stage dysregulation of actin microfilaments is followed by progressive disorganization of microtubules and intermediate filaments at later stages. These stage-specific, step-wise changes provide further insights into the time and spatial sequence of events that lead to the fully transformed state since these changes are also observed in aggressive human ovarian cancer cell lines independent of their histological type. Moreover, our studies support a link between aberrant cytoskeleton organization and regulation of important downstream signaling events that may be involved in cancer progression. Thus, our MOSE-derived cell model represents a unique model for in depth mechanistic studies of ovarian cancer progression.

## Introduction

Ovarian cancer accounts for only 3% of diagnosed cancers, but is the fifth leading cause of cancer deaths among woman, with five-year survival rates of only 45% [1]. The average age of diagnosis is 63 years of age, and most patients (62%) present with metastatic disease at time of diagnosis [1]. Ovarian cancer is a heterogeneous disease with various histo- or clinicopathological subtypes that develop and present differently. The conventional view is that approximately 90% of ovarian cancers are derived from the single-cell layer of surface epithelium that surrounds the ovary [2]. As the ovarian epithelium transforms into a malignant phenotype, it differentiates into several subtypes that have been categorized into serous, mucinous, endometrioid and clear cell carcinoma, based on their morphology rather than their genotype [3]. However, the origin of individual subtypes may vary and a higher contribution from fallopian tubes and the endometrium to more aggressive cancers is currently in discussion [4]. The origin of both ovarian and fallopian epithelial is the same, namely the coelomic epithelium [2] which may contribute to the controversy.

Epithelial ovarian cancers show a high degree of genetic heterogeneity as a result of mutations, silencing, and deletions. Since changes in gene expression, either through mutation, epigenetic regulation, or differential splicing events, influence tumor development, progression, drug responsiveness and ultimately the survival of the patient, the identification of the tumor subtype and its genetic fingerprint is essential. Recently, a new classification of epithelial ovarian tumors into type I and type II cancers has been proposed: type 1 are benign to borderline tumors with relatively stable genotypes while type II includes aggressive and high grade tumors that are genetically instable and exhibit substantial genetic changes [5]. Most epithelial cancers follow a progression scheme in which initiated cells progress to adenomas to adenocarcinomas and metastasis, accumulating genetic alterations in a stepwise manner during progression [6]. This sequence has also been described for low-grade ovarian carcinomas; it is, however, debated if all ovarian cancers follow this cancer development since precursor lesions for the most aggressive ovarian tumors (type II) have not been conclusively identified [5]. Recently, Lee et al. have proposed that the fimbria of the fallopian tube may be the origin for “Type II” serous carcinomas cells [7]. They propose that type II tumors arise from “p53 signature” precursor lesions originating from amplification of secretory epithelial cells. Subsequent mutations then facilitate progression to serous tubal intraepithelial carcinoma and ultimately to serous carcinoma.

Currently, gene expression patterns have only been used successfully to distinguish between mucinous and clear cell from serous carcinomas [8] or between low-grade, low malignant potential and high-grade, metastatic tumors [9], [10], [11]. Reliable molecular or clinical markers to identify changes in the early stages of progression have not been established yet, and since the early stages of the disease are relatively asymptomatic the diagnosis often only occurs at late stages. Therefore, the characterization of gene expression profiles of early stage precursor lesions of ovarian cancer could provide new insights and identify novel targets for preventive and treatment efforts.

We have previously developed and characterized a cell model of epithelial ovarian cancer progression to study the sequence of events that lead to epithelial ovarian cancer [12]. The syngeneic mouse ovarian surface epithelial (MOSE) cells, derived from the C57BL6 mice, have undergone spontaneous transformation in cell culture. The heterogeneous MOSE cells undergo distinct phenotypical changes as they are continuously passaged in culture, with early passages representing a premalignant, non-tumorigenic phenotype, intermediate passages representing a transitional phenotype, and later passages progressing to a highly aggressive malignant phenotype when administered to immunocompetent mice. Transitional states of progression were distinguishable by alterations in growth rates, cell size, loss of contact inhibition of growth, and the capacity to grow as spheroids under non-adherent conditions. Importantly, both the MOSE-I (intermediate passage) and MOSE-L (late passage) cells have also acquired the capacity to form tumors when injected into the peritoneal cavity of syngeneic immunocompetent mice, albeit the former was less invasive [12].

In the present study, we identified significant changes in gene expression patterns as non-transformed MOSE-derived cells transition to more aggressive phenotypes and used gene ontology tools to determine their functional categories. The transitional states of this model allowed us to identify stage-dependent genes, gene products and signal transduction pathways involved in ovarian tumor progression. Here we highlight progressive changes that lead to a highly dysregulated cytoskeleton. Many of these changes were confirmed in archived human ovarian cancer microarray data sets. Importantly, we demonstrate that cytoskeleton disorganization can have profound effects on the subcellular localization of important signaling intermediates, which ultimately may lead to modulated signaling pathways contributing to ovarian cancer development. These genes, their gene products and the associated signaling pathways may represent novel targets for early intervention of neoplastic progression.

## Results

### Differentially regulated genes in mouse ovarian cancer progression

**Figure 1:**
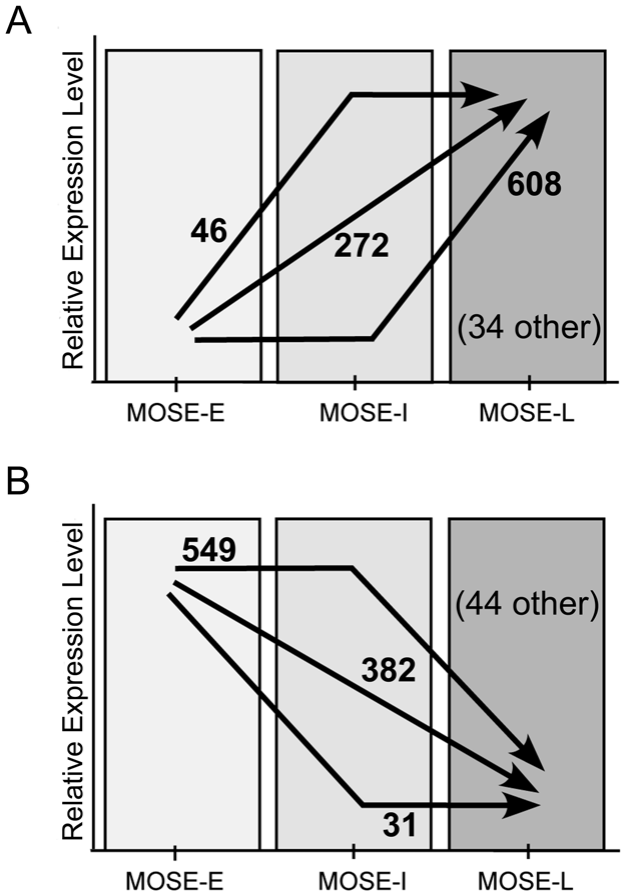
Gene expression changes during progression of MOSE cells. Of 45,102 probe sets analyzed, 970 were significantly (p≤0.05) up-regulated (**A**) and 1006 were down-regulated (**B**) greater than two fold. Arrows indicate pattern of expression changes with number of probe sets indicated next to the arrow. Probe sets indicated as other did not follow the described patterns.

To identify gene expression changes during the progression of epithelial ovarian cancer and determine potential stage-specific patterns, we used whole genome microarray analysis to compare gene expression levels in cells representing benign (MOSE-E), intermediate (MOSE-I), and malignant (MOSE-L) stages of mouse ovarian cancer. Three biological replicates were used to take into account variations within the heterogeneous cultures. Of the 45,102 probe sets on the microarray (representing 18,136 annotated genes), 960 probe sets were found to be significantly up-regulated (701 annotated genes) and 1006 were significantly down-regulated (711 annotated genes) greater that 2 fold (p≤0.05) between MOSE-E and MOSE-L cells. Of these 1966 changing probe sets, 58.9% exhibited no significant change in expression levels during the progression between MOSE-E and MOSE-I, indicating the majority of changes in gene expression are associated with later events in the malignant progression in our model, with 608 increasing and 549 decreasing as cells transition from MOSE-I to MOSE-L. In contrast, 33.3% of the affected genes showed a progressive increase (272 probe sets) or decrease (382 probe sets) in expression as cells transition from MOSE-E to MOSE-I to MOSE-L cells (Figure 1). A small number of affected probes sets, 3.9%, demonstrated MOSE-I/MOSE-E ratios that were within 0.4 fold of MOSE-L/MOSE-E ratios, indicating that these gene expression changes may be associated with very early events in malignant progression of our cells. Together these data indicate that most of the changes in gene expression levels either occur continually, in a stepwise fashion, throughout the progression of our model or take place in later stages while only a limited subset change during early stages. The complete data set can be found in the GEO data base (GSE24789).

### Over-represented gene ontology categories in ovarian cancer progression

To detect pathways that may contribute to the promotion and progression of ovarian cancer, the Gene Trail program was used to identify the functional categories of genes that demonstrate statistically significant changes in their expression levels between MOSE-E and MOSE-L cells. Gene Trail is an advanced gene set enrichment analysis tool that determines over-represented gene ontology categories in data sets [13]. The over-represented cellular component, biological process, and molecular function gene ontology categories found in the MOSE-L versus MOSE-E differentially expressed gene sets are listed in Table 1 (p<0.01). Over-representation of genes in the cell cycle and cell proliferation categories was anticipated due to the previously reported increased growth rate of the MOSE-L cells [12] and the involvement of the uncontrolled cell proliferation in cancer [14]. Interestingly, the cytoskeleton and Metal Ion/Cation binding categories represented a significant number of the differentially expressed genes, with a substantial overlap of genes categorized in both of these ontology categories. However, in contrast to the broad range of functions of the genes in the Metal Ion/Cation binding category, genes compiled in the cytoskeleton gene ontology category were functionally very specific. Since it is thought that changes in the expression levels of cytoskeletal proteins and their regulators are associated with progression and metastasis [15], [16], [17], the changes in genes involved in the structure and regulation of the cytoskeleton during progression of our MOSE model were the subject of further investigation.

**Table 1:** Over Represented Gene Ontology Categories by Differentially Expressed Genes in MOSE Cell Stages Late vs. Early.

Cellular Component	Number of Genes Regulated
Cytoskeleton	141
*Actin Cytoskeleton*	90
*Microtubule Cytoskeleton*	44
*Intermediate Filament cytoskeleton*	7
Metal Ion/Cation Binding	254
Cell Cycle	106
Lipid/Steroid Metabolism	79
Intracellular Transport	71
Cell Proliferation	68
Golgi Apparatus	68
Chromosome	42
Extracellular Matrix	38
Membrane Organization	35
Tyrosine Kinase Receptor Signaling Pathway	30
Lysosome	25
Protein Translation	16
Tyrosine Phosphatase	16
Exonuclease Activity	12
Nuclear Pore	11
Insulin-like Growth Factor Binding	7
Non-G-Protein Coupled 7 TM Receptor Activity	5

Gene Trail program was used to analyze genes that are expressed above background and demonstrate statistically significant changes in gene expression between MOSE-E and MOSE-L cells. Cellular component, biological process, and molecular function gene ontology categories significantly over-represented (p<0.01) are listed.

### Disorganization of the cellular cytoskeleton during malignant progression

#### Actin Cytoskeleton

Of the 141 genes categorized within the cytoskeleton gene ontology category, 90 have gene products that are subunits of actin filaments (Table 2) or are involved in the organization and regulation of the actin cytoskeleton (Table 3; full list in supplemental Table S1). For most of these genes, expression levels gradually changed in a stepwise manner as cells transitioned from MOSE-E to MOSE-I to MOSE-L, indicating that these changes are continuously occurring throughout progression. Only three genes, γ-actin 1, formin 1, and drebrin 1, demonstrated MOSE-I/MOSE-E ratios that were within less than 0.4 fold of MOSE-L/MOSE-E ratios, suggesting these are early changes in malignant progression (Table 2 and 3). Seven genes, including integrin-αv, -β1, and -β2, showed expression levels that changed by the greatest magnitude in MOSE-I cells, two of which were confirmed by qRT-PCR (Table 2 and Table 3). A large number of these genes are dysregulated in cancer or involved in metastasis including all of the 15 genes that were confirmed by qRT-PCR (Table 2 and 3).

**Table 2:** Differentially expressed actin and focal adhesion associated genes in MOSE cell stages.

Gene symbol	Gene Name	Accession Number	I/E	p-val	L/E	p-val
**Actin**						
Acta1	alpha actin 1	NM_009606	−2.2	0.0547	−2.6	0.0487
***Acta2***	***alpha actin 2***	***NM_007392***	***−2.8***	***0.0698***	***−19.3***	***0.0139***
***Actg1*** *	***gamma actin 1***	***NM_009609***	***−2.1***	***0.0331***	***−2.1***	***0.0331***
***Actg2***	***gamma actin 2***	***NM_009610***	***−8.6***	***0.0742***	***−12.6***	***0.0552***
**Focal Adhesion**						
***Actn1***	***actinin, alpha 1***	***NM_134156***	***−3.8***	***0.0454***	***−5.3***	***0.0347***
Fblim1	filamin binding LIM protein 1	NM_133754	−1.6	0.1103	−2.5	0.0282
Itga7	integrin alpha 7	NM_008398	−5.3	0.0179	−7.1	0.0116
*Itgav*	*Integrin alpha V*	*NM_008402*	*−3.7*	*0.0079*	*−1.5*	*0.2389*
*Itgb1*	*Integrin beta 1*	*NM_010578*	*−2.1*	*0.0441*	*1.1*	*0.7088*
Itgb2	integrin beta 2	NM_008404	7.2	0.0059	4.1	0.0365
Itgb5	integrin beta 5	NM_010580	1.7	0.0347	2.7	0.0313
Lasp1	LIM and SH3 protein 1	NM_010688	−1.2	0.2772	−2.7	0.0015
Nck2	non-catalytic region tyrosine kinase adaptor protein 2	NM_010879	1.6	0.0027	2.7	0.0097
Parva	parvin, alpha	NM_020606	−2.0	0.0646	−3.3	0.0314
***Pxn***	***Paxillin***	***NM_133915***	***1.4***	***0.0765***	***2.2***	***0.0094***
Tgfb1i1	TGF beta 1 induced transcript 1	NM_009365	−3.7	0.0668	−49.0	0.0251
***Tns1***	***tensin 1***	***NM_027884***	***−2.3***	***0.0520***	***−5.1***	***0.0162***
***Vcl***	***Vinculin***	***NM_009502***	***−2.4***	***0.0658***	***−3.8***	***0.0355***
Zyx	Zyxin	NM_011777	−3.8	0.0383	−4.5	0.0317

List of genes differentially regulated (fold differences ≥2, p<0.05) which are structural or regulatory proteins of the actin cytoskeleton. Genes in italics were analyzed by qRT-PCR, in bold were validated to change significantly between MOSE-E and MOSE-L cells, and those not in bold were validated to change significantly (p<0.05) between MOSE-E and MOSE-I cells.

*denotes genes that are already changed in MOSE-I and maintain these expression levels in MOSE-L.

**Table 3:** Differentially expressed actin binding regulating genes in MOSE cell stages.

Gene symbol	Gene Name	Accession Number	I/E	p-val	L/E	p-val
Actr3	ARP3 actin-related protein 3 homolog (yeast)	NM_023735	−1.4	0.0095	−2.0	0.0102
***Akap12***	***A kinase (PRKA) anchor protein (gravin) 12***	***NM_031185***	***−9.9***	***0.0151***	***−11.8***	***0.0141***
Anln	anillin, actin binding protein	NM_028390	−1.5	0.0408	−2.5	0.0144
Arhgap24	Rho GTPase activating protein 24	NM_029270	−8.5	0.0096	−39.4	0.0059
Arhgap6	Rho GTPase activating protein 6	NM_009707	2.5	0.1591	12.8	0.0033
Arpc5l	actin related protein 2/3 complex, subunit 5-like	NM_028809	1.7	0.0110	2.5	0.0150
Cap1^+^	CAP, adenylate cyclase-associated protein 1	NM_007598	−2.5	0.0014	−2.1	0.0260
Cdc42ep2	CDC42 effector protein (Rho GTPase binding) 2	NM_026772	1.5	0.2568	−3.1	0.0029
Cdc42ep3	CDC42 effector protein (Rho GTPase binding) 3	NM_026514	−1.8	0.0276	−3.1	0.0063
Cdc42ep5	CDC42 effector protein (Rho GTPase binding) 5	NM_021454	1.1	0.7844	−3.6	0.0013
Dbn1*	drebrin 1	NM_019813	−2.1	0.0034	−2.1	0.0021
***Diap3***	***diaphanous homolog 3 (Drosophila)***	***NM_019670***	***−2.1***	***0.0016***	***−4.2***	***0.0045***
Evl	Ena-vasodilator stimulated phosphoprotein	NM_007965	−1.3	0.1199	−2.6	0.0043
Fmn1*	formin 1	NM_010230	−2.5	0.0280	−2.4	0.0478
Fyn	Fyn proto-oncogene	NM_001122893	1.4	0.1021	2.7	0.0362
***Flnb***	***filamin, beta***	***NM_134080***	***−5.3***	***0.0283***	***−4.0***	***0.0385***
Fscn1	fascin homolog 1, actin bundling protein	NM_007984	−1.5	0.0487	−4.1	0.002
***Gsn***	***Gelsolin***	***NM_146120***	***1.2***	***0.0496***	***2.4***	***0.0284***
***IQGAP2***	***IQ motif, GTPase actinvating protein 2***	***NM_027711***	***3.6***	***0.4092***	***14.0***	***0.0185***
*IQGAP3*	*IQ motif, GTPas- actinvating protein 3*	*NM_178229*	*−2.1*	*0.0140*	*−2.1*	*0.0987*
Ivns1abp	influenza virus NS1A binding protein	NM_001039511	−2.6	0.0134	−2.1	0.0233
Lmo7	LIM domain only 7	NM_201529	−3.7	0.0008	−2.7	0.0104
Map2k1	mitogen activated protein kinase kinase 1	NM_008927	1.4	0.0781	2.2	0.0236
Map2k5	mitogen activated protein kinase kinase 5	NM_011840	1.3	0.1457	2.0	0.0045
Map3k1	mitogen activated protein kinase kinase kinase 1	NM_011945	1.1	0.5470	2.1	0.0028
Marcks	Myristoylated alanine-rich kinaseC substrat	NM_008538	−1.6	0.0059	−2.1	0.0059
***Msn***	***Moesin***	***NM_010833***	***−1.8***	***0.0012***	***−2.4***	***0.0007***
Mtss1	metastasis suppressor 1	NM_144800	1.7	0.2052	−2.2	0.0114
Myh10	myosin, heavy polypeptide 10, non-muscle	NM_175260	−2.4	0.0770	−4.0	0.0264
Myh9	myosin, heavy polypeptide 9, non-muscle	NM_022410	−2.3	0.0567	−2.3	0.0558
Mylip	myosin regulatory light chain interacting protein	NM_153789	−1.1	0.5508	−2.2	0.0042
Myo18a	myosin XVIIIa	NM_011586	1.2	0.0760	2.9	0.0281
Myo1c	myosin IC	NM_001080775	−2.6	0.0165	−3.2	0.0135
Palld	palladin, cytoskeletal associated protein	NM_001081390	−1.9	0.0531	−3.1	0.0145
Plcb1	phospholipase C, beta 1	NM_019677	1.5	0.2930	4.4	0.0015
Plcb4	phospholipase C, beta 4	NM_013829	1.7	0.1110	3.2	0.0165
Rhoj	ras homolog gene family, member J	NM_023275	−2.7	0.0256	−7.5	0.0077
Rhou	ras homolog gene family, member U	NM_133955	1.2	0.3921	2.3	0.0257
Sorbs1	sorbin and SH3 domain containing 1	NM_009166	−28.3	0.0496	−15.4	0.0545
***Tpm2***	***Tropomyosin 2, beta***	***NM_009416***	***−1.9***	***0.2948***	***−42.1***	***0.0191***
Tpm3	Tropomyosin 3, gamma	NM_022314	−1.4	0.1108	−2.3	0.0069
Tpm4	tropomyosin 4	NM_001001491	−1.4	0.1012	−2.4	0.0165
***Wasl***	***Wiskott-Aldrich syndrome-like (human)***	***NM_028459***	***1.0***	***0.7863***	***2.1***	***0.0008***

List of genes differentially regulated (fold differences ≥2, p<0.05) which are structural or regulatory proteins of the actin cytoskeleton. Genes in italics were analyzed by qRT-PCR; genes in bold changed significantly between MOSE-E and MOSE-L cells and those not in bold changed significantly between MOSE-E and MOSE-I cells.

*denotes genes that are already changed in MOSE-I and maintain these expression levels in MOSE-L.

**Figure 2:**
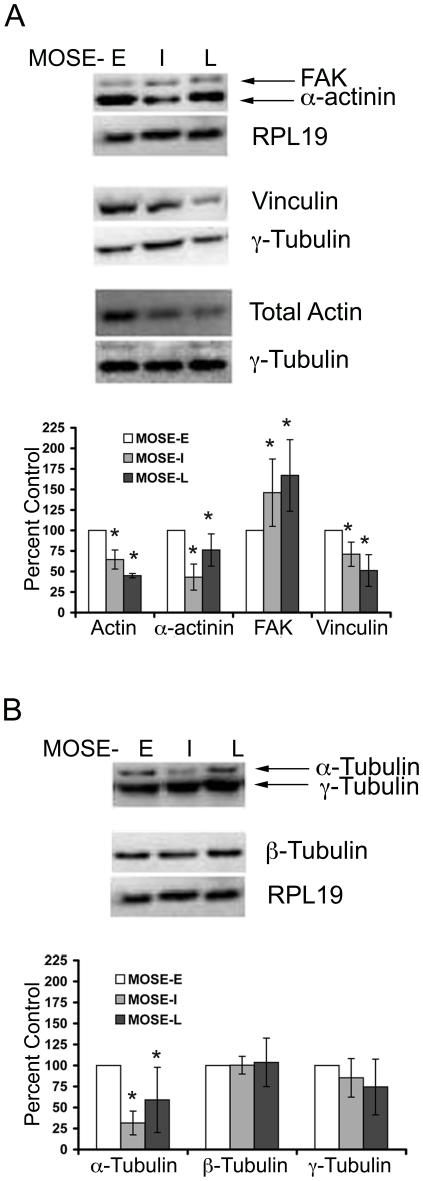
Levels of cytoskeleton and actin regulating proteins in neoplastic progression. Whole cell extracts from MOSE-E (E, white bars), MOSE-I (I, grey bars), and MOSE-L (L, black bars) cells were subjected to Western blot analysis with antibodies directed against (**A**) actin regulating proteins and (**B**) microtubule proteins. Expression levels are expressed as percent MOSE-E levels normalization to ribosomal protein L19 (RPL19) or γ-tubulin for three biological replicates done in duplicate ± the standard deviation. A representative blot from the three biological replicates is shown. *p≤ 0.01.

**Figure 3:**
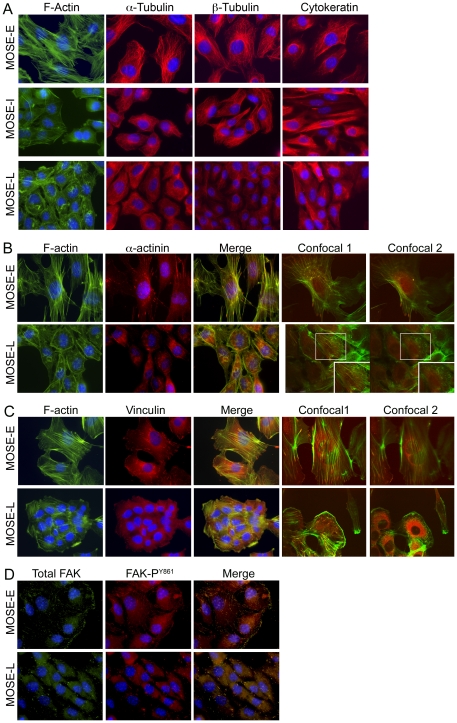
Organization of the cytoskeleton and localization of actin regulating proteins with neoplastic progression. (**A**) Immunofluorescent staining of MOSE-E, MOSE-I and MOSE-L cells to visualize actin filaments (phalloidin, green), α- tubulin (2^nd^ column), β- tubulin (3^rd^ columns), or cytokeratin (4^th^ column) along with the nucleus (blue, DAPI). (**B and C**) Triple staining of MOSE-E and MOSE-L cells with DAPI (blue), phalloidin (f-actin, green), and antibodies against α-actinin (red, B) or vinculin (red, C). The confocal images shown are 0.6 µm apart within the cell, with image 1 starting at the base of the cell and image 2 towards the top of the cell. Co-localization appears as yellow in merged and confocal images. (**D**) Triple staining of MOSE-E and MOSE-L cells with DAPI (blue), antibody against FAK (green), and antibody against FAK phosphorylated tyrosine 861 (red, FAK^Y861^). Yellow in merged image indicates co-localization. (Original magnification X600)

**Figure 4:**
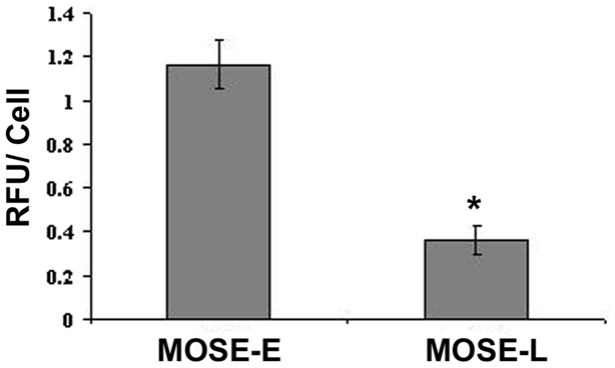
Quantitation of filamentous actin in pre-malignant and malignant MOSE cells. Equal numbers of MOSE-E or MOSE-L cells where plated. After 48 hours, cells were fixed with paraformaldehyde and stained with phalloidin conjugated with Alexa Fluor^488^. The phalloidin was solubilized with MeOH and fluorescence was determined. Data were normalized to cell number and presented as the mean relative fluorescent units (RFU) per cell ± the standard deviation. * p≤ 0.01.

The gene products of a subset of genes confirmed by qRT-PCR were also analyzed by western blot as well as immunofluorescence microscopy to determine potential differences in their subcellular localization. The microarray results indicated a progressive decrease of α-actin and γ-actin mRNA, which was confirmed by qRT-PCR (Table 2), however, no changes of β-actin were observed. This corresponded to a decrease in total actin protein levels during progression (Figure 2A). Furthermore, examination of F-actin architecture by immunofluorescence microscopy revealed distinct differences of the actin subcellular organization. MOSE-E cells exhibited long, well-defined cable-like stress fibers after staining with Alexa Fluor^488^ conjugated phalloidin, whereas the malignant MOSE-L cells displayed less distinct F-actin structures and organization. (Figure 3A, 1^st^ column). In MOSE-L cells, actin structures ranged from small thin stress fibers to prominent “ruffled” zones with very short actin filaments, reminiscent of podosomes (Figure 3A and 3B, confocal image 2 inset). Of note, the MOSE-I exhibited F-actin disorganization similar to that of MOSE-L cells (Figure 3A). To specifically compare cellular F-actin content between MOSE cell lines, a procedure based on fluorescently conjugated phalloidin was employed. As shown in Figure 4, total cellular F-actin was decreased by 78% (p<0.01) in MOSE-L cells compared to MOSE-E cells, confirming qRT-PCR and Western results.

Confocal microscopy revealed large difference in the thickness of the cells; MOSE-E cells had an average thickness of 2 µm, indicating these cells are rather flat when grown on plastic, while MOSE-L cells exhibited an average thickness of 4.4 µm across cytoplasmic regions. The confocal images shown in Figure 3B (confocal image 1 and 2) are consecutive z-stacks 0.6 µm apart starting at the base of the cell where actin fibers are found abundantly at attachment sites. Since the average MOSE-L cell is 4.4 µm thick, the second image captures approximately the middle third of the cell, not the membrane, suggesting the non-fibrous structures in MOSE-L cells are not the result of membrane ruffling. However, they are within the cell cytoplasm and are reminiscent of structures characterized in invasive breast cancer cells as podosomes [18]. In contrast, MOSE-E (Figure 3B) cells showed stress fibers throughout the cells with no short actin filaments.

#### Microtubules

Gene products that compose or regulate the microtubule network comprised the second largest set of genes (44 of 141) affected during neoplastic transformation of MOSE cells (Table 4; full list in supplemental Table S2). All but six genes are only up- or down-regulated in the MOSE-L cells. Five genes (Tubb2b, Cenpe, Mtap6, Ndn, and Vav2) had expression levels that gradually change from MOSE-E to MOSE-I to MOSE-L, indicating that these changes are continuously occurring throughout progression. Only one gene, Ninl, demonstrated MOSE-I/MOSE-E ratios that where within less than 0.4 fold of MOSE-L/MOSE-E ratios, suggesting that this is an early event in malignant progression (Table 4). Interestingly, of the 44 differentially expressed microtubule and microtubule-associated genes, 12 genes encode for proteins involved in chromosome congression (Kif18a, Kif22, Kif4), segregation (Aspm Cenpe, Ckap2, Incenp, Jub, Kif20a, Kif23, Lats2, Prc1), and/or cytokinesis (Incenp, Kif20a, Kif23, Prc1) (Table 4) [19]. All 12 genes exhibited decreased expression with five of the 12 genes coding for kinesins which are molecular motors that use the energy of ATP hydrolysis to move along the surface of microtubule filaments or destabilize them [19], [20].

**Table 4:** Differentially Expressed Microtubule and Microtubule Associated Genes in MOSE cell stages.

Gene Symbol	Gene Name	Accession Number	I/E	p-val	L/E	p-val
**Microtubule**						
Tuba4a	tubulin, alpha 4A	NM_009447	1.5	0.0301	−2.2	0.0146
Tubb2a	tubulin, beta 2a	NM_009450	−1.2	0.4725	−3.5	0.0007
Tubb2b	tubulin, beta 2b	NM_023716	−2.8	0.0200	−3.3	0.0144
Tubb2c	tubulin, beta 2c	NM_146116	1.2	0.2601	−2.1	0.0261
***Tubb3***	***tubulin, beta 3***	***NM_023279***	***1.5***	***0.1902***	***−3.0***	***0.0362***
Tubb6	tubulin, beta 6	NM_026473	−1.2	0.0437	−4.7	0.0013
**Microtubule Binding and Regulation**						
Aspm^+^	asp (abnormal spindle)-like	NM_009791	−1.7	0.0467	−3.0	0.0300
Cenpe^+^	centromere protein E	NM_173762	−2.2	0.0246	−3.7	0.0171
Ckap2^+^	cytoskeleton associated protein 2	NM_001004140	1.1	0.3932	−2.2	0.0324
Ckap2l	cytoskeleton associated protein 2-like	NM_181589	1.0	0.2245	−2.8	0.0050
Ckap4	cytoskeleton-associated protein 4	NM_175451	−1.7	0.0290	−3.0	0.0077
Dnm2	dynamin 2	NM_001039520	1.7	0.2847	4.4	0.0049
Dync1i1	dynein cytoplasmic 1 intermediate chain 1	NM_010063	1.4	0.4035	6.1	0.0450
Incenp^+^	inner centromere protein	NM_016692	1.1	0.1875	−2.1	0.0379
Jub^+^	Ajuba	NM_010590	−3.6	0.0390	−3.2	0.0451
Kif1b	kinesin family member 1B	NM_008441	−1.9	0.0304	−2.3	0.0195
***Kif18a*** ^+^	***kinesin family member 18A***	***NM_139303***	***−1.2***	***0.2028***	***−2.8***	***0.0062***
***Kif20a*** ^+^	***kinesin family member 20A***	***NM_009004***	***1.2***	***0.1513***	***−2.7***	***0.0331***
Kif21a	kinesin family member 21A	NM_016705	1.5	0.2060	2.4	0.0334
*Kif22* ^+^	*kinesin family member 22*	*NM_145588*	*1.0*	*0.2291*	*−2.7*	*0.0178*
***Kif23*** ^+^	***kinesin family member 23***	***NM_024245***	***−1.2***	***0.0970***	***−3.4***	***0.0082***
Kif26b	kinesin family member 26B	NM_001161665	1.0	0.9068	−3.9	0.0102
Kif2c	kinesin family member 2C	NM_134471	−1.2	0.0918	−2.9	0.0211
Kif4^+^	kinesin family member 4	NM_008446	−1.4	0.0966	−2.1	0.0306
Klc1	kinesin light chain 1	NM_008450	−1.4	0.0455	−2.6	0.0009
Klc4	kinesin light chain 4	NM_029091	1.3	0.3611	3.1	0.0015
Lats2^+^	large tumor suppressor 2	NM_015771	−3.4	0.0035	−2.7	0.0058
Mtap6	microtubule-associated protein 6	NM_010837	−3.0	0.0012	−15.7	0.0004
Ndn	necdin	NM_010882	−3.7	0.0124	−26.8	0.0017
Ninl*	ninein-like	NM_207204	−2.6	0.0068	−2.2	0.0029
Pea15a	phosphoprotein enriched in astrocytes 15A	NM_011063	−1.3	0.2698	−2.9	0.0179
Prc1^+^	protein regulator of cytokinesis 1	NM_145150	−1.4	0.0245	−2.6	0.0158
Shroom3	shroom family member 3	NM_015756	−1.2	0.3917	2.8	0.0115
Tbcel	tubulin folding cofactor E-like	NM_173038	1.2	0.1501	3.5	0.0292
Vav2	vav 2 guanine nucleotide exchange factor	NM_009500	−2.1	0.0226	−4.0	0.0062

List of genes differentially regulated (fold differences ≥2, p<0.05) which are structural or regulatory proteins of the microtubule network. Genes in italics were analyzed by qRT-PCR and those in bold were validated to change significantly.

*denotes genes that are already changed in MOSE-I and maintain these expression levels in MOSE-L, + denotes genes that have products involved in chromosome congression, segregation, and/or cytokinesis.

A significant decrease in the levels of α-tubulin isoform 4a and multiple isoforms of β-tubulin were also noted in the microarray data. Confirmation by qRT-PCR of individual isoforms proved difficult because of high levels of homology, but the decrease of tubulin β3 mRNA in MOSE-L cells was confirmed. However, no significant changes of β-tubulin protein levels between MOSE-E and MOSE-L cells were detected (Figure 2B). In contrast, α-tubulin protein levels were decreased by 34% in MOSE-L cells and 67% in MOSE-I cells when compared to MOSE-E levels. No significant changes in γ-tubulin mRNA (data not shown) or protein levels (Figure 2B) were observed and immunostaining revealed no readily discernible differences in protein localization between MOSE-E and MOSE-L cells (data not shown).

Importantly, there were notable differences between MOSE-E and MOSE-L cells when the subcellular organization of microtubule proteins, α- and β-tubulin, were examined by immunofluorescence (Figure 3A). In MOSE-E cells, both α- and β-tubulin appear as long defined filaments radiating from what is likely to be the perinuclear localized centriole (Figure 3A, 2^nd^ and 3^rd^ columns top panel), reported to be a normal organization of tubulin in epithelial cells. In contrast, in MOSE-L cells tubulin filaments were less defined, exhibiting random disorganized branching and the origin of tubulin polymerization was not readily apparent in many cells (Figure 3A, 2^nd^ and 3^rd^ columns, bottom panel). MOSE-I cells appear to have an intermediate phenotype with the centriole apparent in about 50% of the cells along with shorter, less defined filaments than in MOSE-E cells (Figure 3A, 2^nd^ and 3^rd^ column, middle panels).

#### Intermediate Filaments

The final subset of affected cytoskeleton associated genes (7/141) have gene products that make up and regulate the intermediate filament (IF) network. The mRNA levels for number of cytokeratins decreased in MOSE-L cells with cytokeratins 7,8, and 19 verified by qRT-PCR (Table 5). Immunostaining with a pan-cytokeratin antibody revealed that MOSE-E cells have a well organized intermediate filament network extending throughout the cells, whereas the intermediate filament network in MOSE-L cells is composed of short filamentous structures that do not radiate throughout the cell in a organized manner (Figure 3A, last column). Well-defined cytokeratin filaments were noted in only about 25% of MOSE-I cells, with the remainder of cells displaying diffuse cytokeratin staining with the limited organization reminiscent of MOSE-L cells.

**Table 5:** Differentially Expressed Intermediate Filaments and Associated Genes in MOSE cell stages.

Gene Symbol	Gene Name	Accession Number	I/E	p-val	L/E	p-val
**Intermediate Filaments**						
***Krt7***	***keratin 7***	***NM_033073***	***−11.3***	***0.0006***	***−25.8***	***0.0003***
***Krt8***	***keratin 8***	***NM_031170***	***1.2***	***0.0249***	***−2.7***	***0.0035***
Krt14	keratin 14	NM_016958	−3.7	0.0034	−721.9	0.0006
***Krt19***	***keratin 19***	***NM_008471***	***−1.3***	***0.2914***	***−2.2***	***0.0437***
Lmna	lamin A	NM_001002011	−1.4	0.0189	−2.6	0.0043
Lmnb1	lamin B1	NM_010721	−1.8	0.0860	−2.8	0.0581
**Intermediate Filament Binding**						
Eppk1	epiplakin 1, similar to Epiplakin	NM_144848	−6.3	0.0228	2.2	0.0343

List of genes differentially regulated which are structural or regulatory proteins of the intermediate filament network. Genes in italics were analyzed by qRT-PCR and those in bold were validated to change significantly.

### Comparison to archived human ovarian cancer microarray data sets

In order to determine the relevance of the observed changes in the cytoskeleton gene expression levels of our MOSE cell progression model to human ovarian cancer, we evaluated archived DNA microarray data sets which compared gene expression levels in different established human ovarian cell lines with normal ovarian surface epithelial cells as reference (see Materials and Methods for a description of cell lines evaluated). Although differential expression of cytoskeletal genes were not a focal point in these human studies, approximately 50% of the actin and focal adhesion associated genes listed in Table 2 as significantly down-regulated during MOSE cell progression were also significantly down-regulated in the human ovarian cell lines. As shown in Table 6, there was a clear enrichment for significant changes in the actin and focal adhesion associated genes. Using the cumulative bionomial distribution, the estimated probability of observing this many differentially expressed actin and focal adhesion genes in the human studies by chance were 2.23×10^−6^ and 1.87×10^−7^, respectively, for the comparison with data from Nagaraja et al. [21] and Iorio et al. [22]. In addition, comparative analysis revealed that several additional actin binding genes listed in Table 3 were significantly downregulated in the human ovarian cancer cell lines. Of note, Marcks and Tpm2 were downregulated by 10- and 23-fold respectively in aggressive ovarian tumor cells compared to normal OSE. The overlap of differentially expressed genes in the microtubule functional category did not reach significance [21]. This may be a result of the comparatively small changes in gene expression levels in this category. However, the Ndn gene, which was 27 fold down-regulated in the MOSE cells, was as much as 125 fold down-regulated in the human cancer cell lines [21]. Together, these results suggest that changes in the cytoskeleton are common to many ovarian cancer cell lines independent of their histological type.

**Table 6:** Comparison of differentially expressed cytoskeleton and regulatory genes with archived array data sets comparing established human ovarian cell lines with normal ovarian surface epithelium.

Gene	Illumina Rank^a^	Fold change^b^	p-value	Affymetrix Rank^a^	Fold change^b^	p-value
ACTA1	x		x	x		x
ACTA2	1.8%	−7.7	2.00E-06	0.6%	−58.8	5.00E-05
ACTG1	5.7%	−1.8	6.00E-05	x		x
ACTG2	x		x	x		x
ACTN1	2.7%	−2.6	5.00E-06	0.5%	−4.0	2.00E-05
FBLIM1	1.4%	−2.3	8.00E-07	0.8%	−8.3	9.00E-05
ITGA7	x		x	5.3%	−5.0	2.00E-02
ITGAV	x		x	4.8%	−2.4	1.00E-02
ITGB1	x		x	0.8%	−5.3	1.00E-04
ITGB2	x		x	x		x
**ITGB5**	**1.6%**	**−6.3**	**1.00E-06**	**0.7%**	**−4.5**	**5.00E-05**
LASP1	x		x	x		x
MARCKS	0.5%	−10.2	3.00E-08	1.4%	−7.1	5.00E-04
NCK2	5.5%	−1.9	6.00E-05	x		x
PARVA	9.4%	−1.8	3.00E-04	2.9%	−4.5	4.00E-03
PXN	x		x	x		x
TGFB1I1	7.3%	−6.7	1.00E-04	x		x
TNS1	7.8%	−2.9	2.00E-04	4.3%	−7.1	1.00E-02
TPM2	0.03%	−23.1	1.00E-11	1.9%	−7.0	1.00E-03
VCL	8.1%	−2.0	2.00E-04	x		x
ZYX	8.7%	−1.9	2.00E-04	1.3%	−3.6	4.00E-04
NDN	0.5%	−124.6	3.00E-11	0.3%	−23.7	5.0E-06
Binomial probability	1.86E-07			1.04E-08		

The expression levels of genes changed in the MOSE model were compared to changes determined in established human cell lines reported by Nagaraja et al. [21](Illumina data sets) and Iorio et al. [22](Affymetrix data sets). x denotes non-significant changes, or expression levels below detection limit. Only ITGB5 levels changed in the opposite direction than the MOSE cells. a) Rank refers to the percentile rank when the human microarray data sets are sorted by increasing p value. b) Mean Fold change in gene expression of cancer cell lines compared to normal human OSE data sets.

### Changes in actin cytoskeleton regulation and architecture during neoplastic progression

To determine the mechanisms of cytoskeletal deregulation during MOSE malignant progression, we investigated the expression levels and subcellular localization of several regulatory proteins, including α-actinin, vinculin and focal adhesion kinase (FAK). These proteins were chosen because of their involvement in cytoskeleton regulation, cell motility, and cancer progression/metastasis. α-actinin is involved in actin bundling by cross-linking actin filaments and is part of the focal adhesion complex that links the actin cytoskeleton to integrins [23], [24]. The microarray results indicated progressively decreasing α-actinin expression levels which were confirmed by qRT-PCR (Table 2). α-actinin protein levels were significantly decreased in both MOSE-I and MOSE-L cells compared to MOSE-E cells (Figure 2A). A distinct co-localization of α-actinin (red) with actin filaments (green) running parallel to the leading edge was always readily apparent in MOSE-E cells (Figure 3B). In MOSE-L cells, α-actinin appeared largely as diffuse staining in the cytoplasm with considerably less evident co-localiziation with actin filaments (Figure 3B, red). This was also observed in MOSE-I cells (data not shown). Confocal microscopy indicated α-actinin did not co-localize to the very short actin filaments and disorganized actin found in MOSE-L cells (Figure 3B, confocal images and inset).

In addition to actin filament bundling, α-actinin acts as a platform to mediate protein-protein interactions including those involved in forming and maintaining focal adhesions [23], [24]. MOSE cells had variable levels of gene products known to associate with or modulate focal adhesions (Table 2, Focal Adhesions). Also, a number of gene products directly associate with α-actinin to modulate focal adhesions (zyxin, vinculin, integrin b1 and b2) or regulate actin (palladin and syndecan). Changes in mRNA levels of several of these genes were confirmed by qRT-PCR (Table 2). Importantly, genes associated with cancer progression (i.e., Itgb2, Itgb5, paxillin, fyn) displayed increased expression, whereas those thought to suppress progression (i.e., vinculin, gravin) exhibited decreased levels of expression compared to MOSE-E cells.

Vinculin, which binds actin and is part of the focal adhesion complex linking actin to integrins, exhibited both reduced mRNA (Table 2) and protein levels (Figure 2A) during malignant progression. To visualize potential alterations in subcellular localization, MOSE cells were immunostained for both F-actin and vinculin (Figure 3C). In MOSE-E cells, vinculin co-localized to the ends of actin bundles, forming well-defined focal adhesion structures similar to that observed for non-transformed epithelial cells. In contrast, vinculin staining was largely diffuse and only marginally co-localized with actin fibers in the MOSE-L cells. Inherently, the focal adhesion-like structures in MOSE-L cells were less defined and more punctate. Confocal microscopy revealed that vinculin was distributed throughout the cytoplasm of MOSE-L cells and did not appear to associate directly with the disorganized actin, (Figure 2C, confocal images). Similar vinculin staining patterns were observed in 90% of the MOSE-I (data not shown), suggesting that aberrant vinculin subcellular localization is an early event as cells transition from MOSE-E to MOSE-I.

The primary component of focal adhesions, FAK, did not exhibit significant changes in mRNA levels during MOSE progression (Table 2). However, FAK protein levels were significantly elevated in both MOSE-I and –L cells compared to MOSE-E (Figure 2A). To determine if there is also a change in FAK activity and localization, MOSE cells were immunofluorescently stained for total FAK (red) and FAK phosphorylated on tyrosine861 (FAK-P^Y861^, green) (Figure 3D). Of note, phosphorylation of FAK on tyrosine Y^861^ by Src, one of two residues phosphorylated by Src, contributes to cell migration [25], [26]. As shown in Figure 3D, FAK was only marginally associated with the membranes of MOSE-L cells compared to the bright punctate staining at the cell periphery of MOSE-E, but was rather diffusively distributed throughout the cytosol. Overall, there was very little punctate staining of FAK at the periphery of MOSE-L cells. Interestingly, the peripheral total FAK co-localized with the active FAK-Y^861^, suggesting that peripheral FAK is active in both MOSE-E and –L cells (Figure 3C, merge). Since FAK staining requires MeOH fixation, confocal microscopy did not provide conclusive results as to the co-localization of diffuse total FAK and pFAK-Y^861^ observed in MOSE-L cells. Thus, it is unclear whether diffuse pockets of disorganized actin and total FAK contribute to the reduced formation of focal adhesions observed in MOSE-L cells.

### Neoplastic cytoskeleton changes influence signal transduction pathways

The cytoskeleton plays an important role in tumor cell progression and events such as migration and invasion, allowing the cells to adapt and survive in different microenvironments; compounds that regulate cytoskeleton organization have been used as cancer therapeutics [27]. On the other hand, the organization of the cytoskeleton affects cellular organization, adhesion complexes and polarity, and vesicular transports. As noted above, the subcellular localization of proteins associated with focal adhesions displayed aberrations concomitant with the disorganized state of the cytoskeleton. This may allow the tumor cells to bypass cellular homeostatic control mechanisms by diverting signaling proteins to different locations, thereby changing the availability of binding partners or substrates, which may modify signal transduction pathways. Since aberrant signaling is a sign of malignancy [28], immunostaining for global tyrosine and serine phosphorylated proteins was used as a general gauge of signal transduction pathway organization and function.

**Figure 5:**
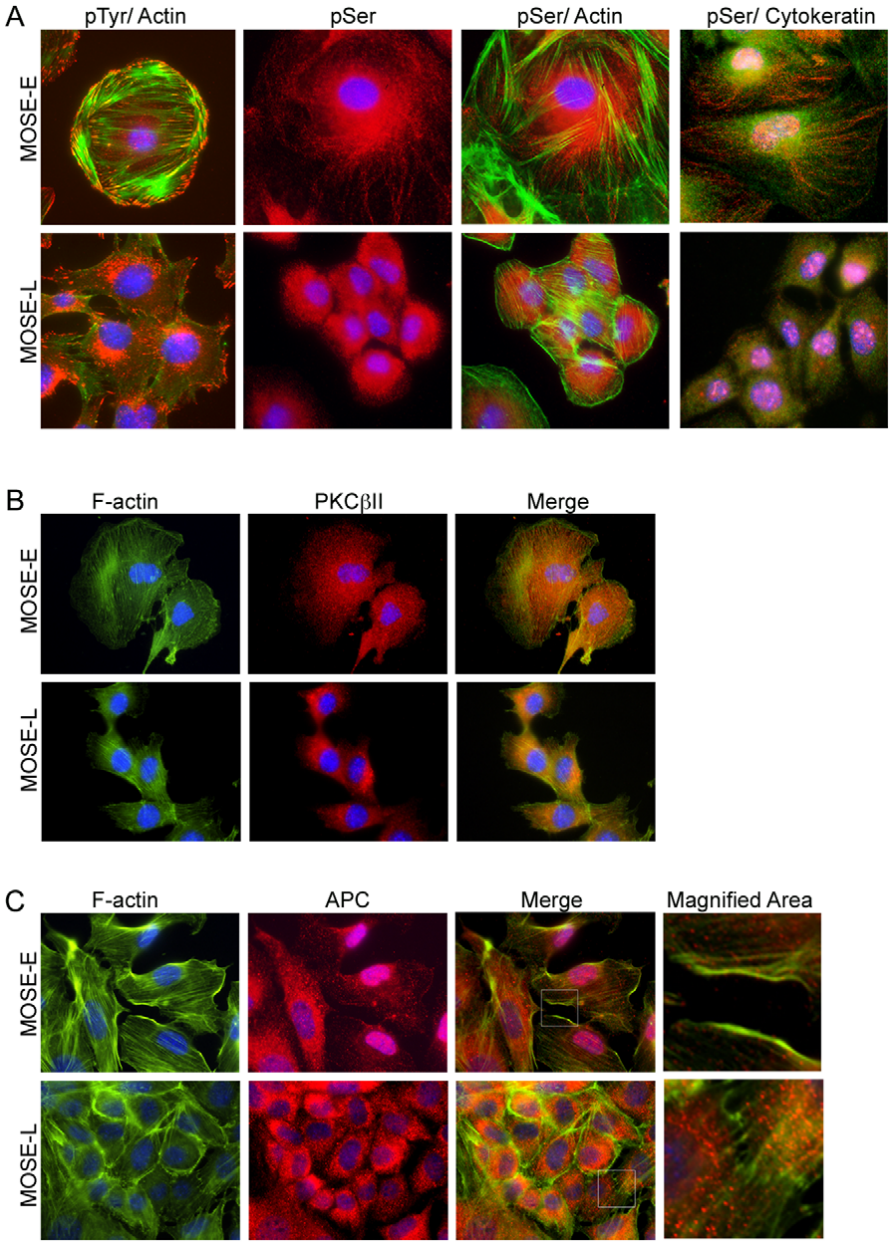
Influence of actin disorganization on localization of signaling proteins PKCβII and APC. (**A**) Immunofluorescent staining for DAPI (blue), phosphotyrosine (red, pTyr), and phalloidin (green, f-actin) or DAPI (blue), phosphoserine (red, pSer), with either phalloidin (f-actin, green) or cytokeratin (green). (**B and C**) Triple staining of MOSE-E and -L cells with DAPI (blue), phalloidin (f-actin, green) and PKCβII (red, B) or APC (red, **C**) antibodies. (Original magnification X600).

Tyrosine phosphorylation, an indicator of receptor and non-receptor tyrosine kinase activity, plays a critical role in cancer cells, regulating proliferation, differentiation, and metabolism; 51 of the 90 tyrosine kinases have been implied in cancer (see recent review [28]. As shown in Figure 5A (top panel), MOSE-E cells showed a distinct phospho-tyrosine staining pattern highly reminiscent of focal adhesions at the cell periphery, with prominent co-localization evident at the ends of actin fibers and only marginal staining in the cytosol. In contrast, phosphotyrosine immunostaining did not co-localize strictly with actin fiber ends, presumably focal adhesions, in MOSE-L cells and was also readily apparent in the cytosol and in perinuclear regions (Figure 5A, bottom panel). Phosphoserine immunostaining, an indicator of downstream signaling and G-protein coupled receptor activity, appeared as organized punctae along filament-like structures radiating from the nucleus in MOSE-E cells. These did not co-localize with actin or cytokeratin; although the staining pattern suggested a co-localization with tubulin, this could not be confirmed since our tubulin and phosphoserine antibodies are produced in the same species, not allowing for double staining (Figure 5A, top panel). In MOSE-L, immunostaining for phosphoserine also appeared as punctae but were less organized (Figure 5A, bottom panel). As expected due to its role in the regulation of the splicing machinery, phosphoserine staining was detected in the nuclei of both MOSE-E and MOSE-L cells.

**Figure 6:**
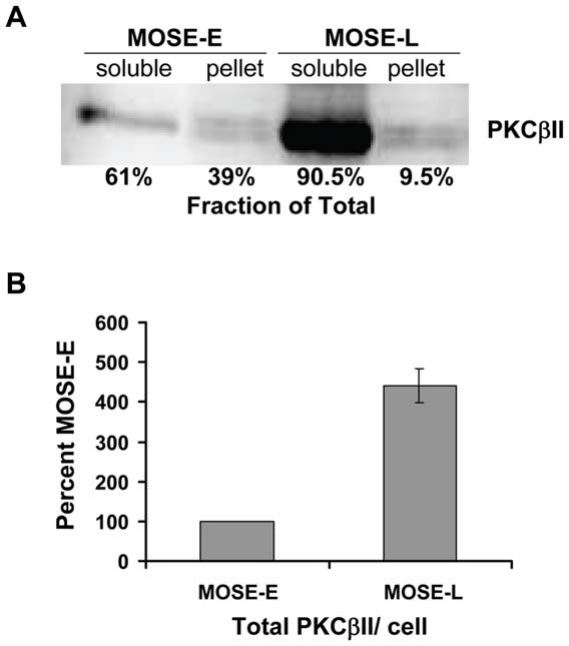
PKCβII protein levels and interactions with cytoskeleton. Equal amounts of protein from MOSE-E and MOSE-L was fractionated into 1% triton X-100 soluble and non-soluble (pellet) portions and analyzed by western blot analysis (**A**). Protein levels are the mean of three measurements expressed as percent of the total PKCβII protein with standard deviations ≤1.5% for all samples, normalized by cell number. (**B**) Total protein levels (soluble + pellet) (mean of three measurements) are expressed as percent of MOSE-E levels. * p<0.001.

To further explore the hypothesis that the actin/tubulin cytoskeleton changes during the progression of MOSE-E to MOSE-L cells could lead to an incorrect localization/scaffolding of proteins and change signal transduction pathways, we analyzed integral signaling proteins that have also been implied in ovarian cancer development: protein kinase C β II (PKCβII) and adenomatous polyposis coli (APC). PKCβII is a member of the serine/threonine kinase family with a broad spectrum of intracellular targets and, thus, a central signaling intermediate in a multitude of signaling pathways. PKCβII is involved in the regulation of proliferation, apoptosis but also promotes angiogenesis, invasion and progression [29], [30]. In MOSE-E cells, PKCβII (Figure 5B, red) appeared as distinct punctae throughout the cytoplasm, co-localizing with actin stress fibers and actin at the leading edge (Figure 5B, merge). In contrast, PKCβII in MOSE-L cells (Figure 5B, bottom panel) was more diffuse and rarely co-localized with actin fibers (specific images of cells showing actin fibers were chosen). PKCβII immunostaining in MOSE-I cells displayed a mixed pattern with commonalities between that observed for both MOSE-E and MOSE-L cells (data not shown). To further investigate this observation, cell fractionation was performed to analyze PKCβII association with cytoskeletal components. Total PKCβII levels increased more than 4-fold in MOSE-L compared to MOSE-E cells (p<0.001) (Figure 6B). This correlates well with the role of overexpressed PKCβII in cancer progression which has led to the development of specific PKCβII inhibitors that alone or in combination with conventional drugs suppressed ovarian cancer cell growth [31]. The percentage of total PKCβII in the cytoskeletal fraction changed from 39% in MOSE-E cells to 9.5% in MOSE-L cells (Figure 6A).

We next investigated the expression and localization of adenomatous polyposis coli (APC), a scaffolding protein that regulates β-catenin metabolism [32] but is also involved in a broad range of other, β-catenin- independent processes such as regulation of microtubule assembly and bundling [33]. APC mutations contribute significantly to colon carcinogenesis [34] but also have been implied in ovarian cancer development [35], [36]. Approximately 50% of MOSE-E cells showed substantial APC staining in the nucleus while only a small percentage of MOSE-I cells and none of the MOSE-L cells displayed nuclear staining. Cells with nuclear APC showed very little cytosolic staining (Figure 5C). APC appeared punctuated in the cytosol of all MOSE cells (Figure 5C, red). Most of the cytosolic APC in MOSE-E and MOSE-I cells appeared to co-localize with actin fibers especially on the cell periphery (figure 5B, top panel); however, this was not observed in MOSE-L cells.

## Discussion

In the present study, we have identified genes and their functional categories that were altered as MOSE-derived cells transition from an early, pre-malignant, to a highly malignant stage. Our gene expression profiles from the transitional stages of MOSE cells displayed statistically significant changes in cell cycle, proliferation, metabolism and other functional categories that corresponded well with many of the morphological changes and biological behaviors observed in our progressive MOSE model, including the loss of contact inhibition, resistance to anoikis, the ability to form colonies in soft agar, and the capacity to form invasive tumors *in vivo* in an immunocompetent host [12]. While many of the gene expression changes may be applicable to other cancer models, the early stage dysregulation of the cytoskeleton architecture as described here highlights its role in early ovarian cancer progression.

Together, our data reflects many of the changes observed in established human ovarian cancer and further identifies several early events involved in neoplastic progression that may represent early targets for therapeutic intervention.

The substantial gene expression changes in the cytoskeleton category led us to focus on both key cytoskeleton proteins and their regulators to further delineate their global role in neoplastic transformation. Of note, stepwise dysregulation of the cytoskeleton has not been studied in depth for the early events in ovarian cancer. During the malignant progression of the MOSE cells, the microfilament, microtubule and intermediate filament systems became sequentially disorganized, highlighted by i) distinct protein level changes, ii) the significant loss of polymerized F-actin, and iii) the decreased capacity for formation of focal adhesions. Interestingly, the global subcellular distribution pattern of proteins phosphorylated on serine or tyrosine residues changed as MOSE cells progressed to a more malignant state, suggesting that signaling pathways were also becoming progressively altered. This may be partly due to the aberrant subcellular localization of proteins resulting from changes to cytoskeletal architecture. In support of the latter, changes affecting PKCβII and APC expression and localization, integral signaling intermediates associated with cancer development, correlated well with cytoskeleton alterations.

### Cytoskeleton and cytoskeleton-regulating proteins

Actin filaments, along with their associated proteins, are essential for cellular morphology, motility and migration, phagocytosis, vesicular movement, cytokinesis, and molecular transport between the plasma membrane and the nucleus [15]. Changes affecting actin cytoskeleton architecture were early events in the transitional progression of our MOSE-derived cells as most of the changes in gene expression and protein levels were readily evident in the MOSE-I cells, an intermediate transitional stage that already has acquired limited tumor-forming capacity. Microtubule organization, essential for cell division, cell migration, vesicle transport and cell polarization [37] was altered progressively during malignant progression culminating in a highly disorganized state in malignant MOSE-L cells. Of particular note is that the observed cytoskeletal alterations during MOSE cell progression have also been reported in several established human ovarian cancer lines that represent late stage aggressive disease. A comparison of our results to microarray data sets from established human ovarian cancer cell lines and benign or normal OSE as a reference [21], [22] demonstrated a highly significant overlap in the changes of cytoskeleton and regulatory genes (see Table 6). Furthermore, in a recent proteomic study of human ovarian cancer, 21 of the 37 proteins found to be differentially expressed between low- and high-grade ovarian cancer cell lines (TOV-81D and TOC-112D, respectively) were involved in cytoskeleton organization, cell adhesion and motility [38]. Similar changes in genes overrepresented in these functional categories were observed by proteomic comparison of several established and widely used ovarian cancer cell lines; it was concluded that these gene changes were not associated with a specific sub-type of ovarian cancer but rather with the cells' aggressive and invasive behavior [39]. Importantly, our data reveal a stepwise accumulation of genetic changes affecting the actin cytoskeleton that are not readily apparent when analyzing human ovarian cancer samples, which are largely representative of late stage disease. Together, these data suggest that the changes in the actin cytoskeleton are a common event in ovarian cancer cells and not restricted to a specific sub-type of ovarian cancer. Thus, these genes and gene products may represent potential early targets for chemotherapeutic intervention against several types of ovarian cancer.

Reciprocal or coordinated regulation of cytoskeleton components, specifically microtubules and the actin cytoskeleton, is becoming more apparent [40], [41], [42]. Our data demonstrating early, more drastic changes in the actin cytoskeleton validate these observations and suggest that the early disorganization of the actin cytoskeleton may be a key element that facilitates further dysregulation of the cytoskeleton in ovarian cancer. Hence, actin and its regulatory and associated proteins may be better therapeutic targets in ovarian cancer. This hypothesis is supported by recent observations demonstrating that interference with actin dynamics is more effective than microtubule disturbance in inhibiting human ovarian cancer cell motility [43], and stabilization of the actin cytoskeleton can be achieved by re-introduction of actin-binding proteins such as calponin [44]. Interestingly, calponin re-expression in ovarian cancer cells also significantly reduced peritoneal dissemination [45].

Prominent stress fibers have been demonstrated in more stationary cells and are thought to inhibit motility, whereas changes in cytoskeleton regulatory proteins have been closely associated with increased cell motility and invasion [46]. Our studies show the sequential loss of stress fibers during MOSE progression. This may be associated with the aberrant expression and localization of cytoskeleton regulators such as vinculin, FAK, and α-actinin, since these regulators form complexes with other membrane proteins such as integrins that together generate signals to regulate proliferation and migration of normal and tumor cells [26], [47]. We have reported the increase in cell proliferation during MOSE progression [12] that correlates well with the changes in the cytoskeleton architecture. Of note, the aberrant expression of α- and β-tubulin, keratin 7, and other cytoskeleton regulators has been reported in drug-resistant ovarian tumors [48], indicating that dysregulation of the cytoskeleton may also contribute to multi-drug resistance. Interestingly, FAK inhibition augmented docetaxel-mediated apoptosis in ovarian cancer cells [49], [50], suggesting that the effects of the cytoskeleton and its regulators are not limited to regulation of cell morphology, adhesion and motility. Thus, the cytoskeleton and its regulators -especially of the actin cytoskeleton in early stages- may be effective chemotherapeutic targets as has been already shown for the microtubule system [27], [51].

It should also be noted that additional actin-binding proteins (see Table 3) such as tropomyosin 2 were found to be significantly down-regulated in MOSE-L cells. Though tropomyosin function is less defined in non-muscle cells, an increase in actin stiffness, protection from branching due to cofilin activity, and formation of lamellipodia has been reported (see recent review [52]). In cancer, frequent changes in tropomyosin expression levels have been noted and loss of tropomyosin has been associated with the switch from a dormant to rapidly growing tumor [53]. Down-regulation of tropomyosin 2 via epigenetic silencing in human ovarian cancer has been reported [54] and recent results in our laboratories using 5′aza deoxycytidine treatment suggest that tropomyosin 2 as well as α-actinin and vinculin are epigenetically silenced in MOSE-L cells (unpublished observations). We have already demonstrated that promoter methylation of the E-cadherin gene results in its silencing during MOSE progression [12]. Future studies will help define at what stage this epigenetic silencing of actin regulatory genes occurs and if these specific genes are potential targets for chemotherapeutic interventions.

### Signal Transduction

Post-translational modifications including protein phosphorylation determine cellular responses and functions. Changes in the equilibrium of the antagonistic kinase and phosphatase activities, especially on tyrosine residues, have been described in many cancers as a result of the oncogenic activation of receptor or non-receptor tyrosine kinases or the inhibition of protein tyrosine phosphatases (e.g., EGFR, Her-2neu, Src, Abl, PTPs) [28]. Changes in G-protein coupled receptors affect the phosphorylation of serine residues and subsequently a multitude of signaling pathways. An increase of tyrosine phosphorylated proteins and altered intracellular localization of both tyrosine or serine phosphorylated proteins during the progression in our MOSE model suggest the relocalization of signaling intermediates may be associated with changes in cellular properties and functions. While it was not within the scope of this study to identify these proteins and characterize affected signaling pathways and downstream events, we have identified an aberrant expression and localization of two important signaling molecules, PKCβII and APC.

PKCβII is critically involved in cancer of several organs including the ovaries [9], [29]. Upon activation, PKCβII is translocated to the membrane and pericentrosomal regions [55], [56] which requires the presence of a well-organized actin cytoskeleton [57]. PKCβII can directly bind to actin, which in turn modulates its substrate specificity via determination of substrate proximity [58], suggesting that the actin cytoskeleton controls the target substrate and, therefore, the regulated signaling pathways [57]. One could speculate that the overexpression and sequestration of activated PKCβII during neoplastic progression provides a survival mechanism, or its proximity to other signaling components may serve to provide the cell with a constitutive endogenous signaling compartment, stimulating cell survival, migration and invasion. The overexpression and pericentrosomal aggregation of PKCβII observed in MOSE-L cells concurrent with actin microfilament disorganization, taken together with previous findings, suggests that the two events may be inherently linked.

Progression to the MOSE-L stage in our model was accompanied by the presence of podosome-like structures throughout the cytoplasm of the cell. PKC activation is associated with the formation of podosomes, which may be immature forms of invadopodia [18], [59]. It also modulates the distribution of F-actin and can lead to a dissociation of vinculin from focal adhesions in transformed cells [60]. Hence, the podosomes-like structures observed in MOSE-L cells may be the indirect result of over-expressed or sequestered PKCβII, but this needs to be investigated further.

Concurrent with the actin cytoskeleton disorganization, aberrant localization of APC was observed during progression to the malignant MOSE-L phenotype. APC serves as a negative regulator of Wnt signaling, acting as a key tumor suppressor gene that is often mutated in colon cancer [34] but has also been implicated in ovarian cancer development [36]. APC is a multifunctional protein, influencing both microtubule assembly and bundling [61] as well as actin polymerization and cell polarity [62]. Recent studies suggest that APC may act in a more regulated fashion by i) direct association with microtubules [63], ii) binding cytoskeleton regulating proteins including IQGAP1 [62], [64] and iii) interacting with intermediate filaments [65], all of which suggest that the cytoskeletal architecture is critical for APC localization [66]. Thus, the early changes in the cytoskeleton in our MOSE cell system may have a direct impact on the subcellular localization of APC influencing its function. Interestingly, in normal colon cells, APC is strongly localized in the nucleus while appearing increasingly in the cytoplasm in colon carcinoma [34]. APC shuttles between nucleus and cytoplasm, sequestering β-catenin to induce degradation in the cytoplasm or dampen β-catenin mediated transcriptional activity in the nucleus [67]. However, the binding to DNA, base excision DNA repair proteins, and phosphotyrosine phosphatases indicates other, yet to be determined functions of APC in the nucleus. The loss of full-length APC activates a DNA demethylase in colon cells and increased the expression of genes that maintain an undifferentiated cellular state [68]. These observations together with the loss of APC during progression of our MOSE-derived cells strongly support a tumor-suppressing effect of nuclear APC.

In summary, gene expression profiling during neoplastic progression of MOSE cells revealed that cytoskeleton associated genes were significantly impacted as cells transitioned from a benign to a malignant stage. Distinct actin regulatory genes were dysregulated at early stages in ovarian cancer progression with microtubule and intermediate filament alterations following at later stages. Our data support the concept of cross-talk between actin, tubulin and intermediate filament regulatory mechanisms. We provide further evidence that progressive disruption of the cytoskeleton architecture plays a pivotal role in subcellular organization of signaling intermediates, particularly with respect to coordinated signal transduction events. Thus, cytoskeleton dysregulation may influence trafficking of proteins and vesicles within the cell, changing the proximity of substrates and enzymes that subsequently lead to aberrant downstream signaling pathways and cellular responses. Finally, our data supports the hypothesis that structural rearrangements of the cytoskeletal architecture are crucial for neoplastic progression, conveying signals from the extracellular matrix to the nucleus that allow cancer cells to adapt to their microenvironment via transcription factor activation and subsequent change of gene expression (see recent review [69]). Many of the changes observed in the present study are also found in human ovarian cancer and therefore validate the use of our model for future mechanistic studies to further define how cytoskeletal organization modulates the subcellular localization of cancer promoting signaling pathways.

## Materials and Methods

### Cell Culture

The MOSE cell model utilized in this study was developed and characterized as previously described [12]. MOSE cells were classified into early (MOSE-E, passages 5–20), intermediate (MOSE-I, passages 60–80), and late (MOSE-L, passages 120–180) stages based on ranges of passage number that displayed similar growth rates, anchorage independent growth efficiencies in soft agar, *in vivo* tumor formation, and aneuploidy. MOSE cell lines were routinely maintained in DMEM high glucose medium (Invitrogen, Carlsbad, CA) supplemented with 4% fetal bovine serum (Hyclone, Logan, UT), 100 mg/ml each of penicillin and streptomycin, 5 mg/ml insulin, 5 mg/ml transferrin, and 5 ng/ml sodium selenite (Invitrogen, Carlsbad, CA). For RNA and protein collection, cells were seeded in 100 mm dishes at 0.5-2×10^6^ cells and grown for 1–2 days (60–80% confluency).

### Gene Chip Micoarrays and Data Analysis

Biological replicate RNA samples for early (passage 13, 14, and 15), intermediate (63, 71, and 73), and late (136, 142, and 143) passages were isolated using the RNeasy Kit according to the manufacturers instructions (Qiagen, Valencia, CA) and treated with ribonuclease-free deoxyribonuclease I (Qiagen, Valencia, CA). The RNA samples were submitted to the Virginia Bioinformatics Institute (VBI) Core Laboratory Facility (CLF) gene expression unit for microarray analysis. At VBI CLF the RNA samples were assayed on the Agilent 2100 BioAnalyzer for qualitative assessment and quantification. cRNA was hybridized to GeneChip Mouse Genome 430 2.0 Arrays (Affymetrix, Santa Clara, CA) containing 45,102 oligonucleotide probe sets representing over 18,000 known genes. We utilized MicroArray Suite 5.0 (Affymetrix, Santa Clara, CA) to process raw microarray data. Data values were normalizes to a trimmed mean of 500 units to allow inter-GeneChip comparisons. Excel Spreadsheet software (Microsoft, Silicon Valley, CA) was used to obtain fold change and and t-test p-values for the pairwise comparisons. After filtering for a maximum signal intensity greater than 500 fluorescent units and significant differences between early and late passages of greater than 2 fold (p≤0.05), data was analyzed for over-represented gene ontology categories using the Gene Trail Program [13], [70](http://genetrail.bioinf.unisb.de/index.php) and Onto-tools Pathway Express (http://vortex.cs.wayne.edu/projects.htm#Onto-Express) [71], [72]. Comparison of MOSE cells with human gene expression data was performed using the Gene Expression Omnibus (GEO) Illumina microarray data sets for a) Normal OSE cells and 10 ovarian cancer cell lines (OVAS, SMOV-2, KK, OVSAYO, RMG-1, OVMANA, OVISE, TOV-21G, ES-2, and OVTOKO) Accession number GSE16568 [21] and b) Affymetrix microarray data sets using Normal OSE cells and 6 additional ovarian cancer cell lines (SKOV3, OVCAR3, OVCA432, OVAW42, IGROV1, and CABA) Accession number GSE19352 [22].

### Real-time Polymerase Chain Reaction PCR (qRT-PCR)

Total RNA was extracted from biological replicate samples as described above. 500 ng of total RNA was reverse-transcribed using the ImProm-II Reverse Transcription System (Promega, Madison, WI) with random hexamer and oligo-dT primers according to the manufacturer's instructions. Quantitative Real-time Polymerase Chain Reaction (qRT-PCR) was performed on 5 ng of cDNA using gene specific primers designed using Beacon Design software (Palo Alto, CA) and SensiMix Plus Sybr mastermix (Quantace, Taunton, MA) in a 15 µL reaction volume. qRT-PCR was performed for 42 cycles at 95°C for 15 seconds, 56–58°C for 30 seconds, and 72°C for 15 seconds, preceded by a 10 minute incubation at 95°C, on the ABI 7900HT Fast Real-Time PCR System (Applied Biosystems, Foster City, CA). Melt curves were performed to insure fidelity of the PCR product. The ΔΔCt method [73] was used to determine fold difference and the student T-test was utilized to ascertain significance.

### Cell Fractionation

Cells were grown in 100 mm tissue culture dishes as described above and cell fractionated essentially as described by Blobe et al. [58]. Briefly, cells were washed in PBS and lysed in 1% Triton X-100 solubilization buffer (15 mM Tris, pH 7.5, 120 mM NaCl, 25 mM KCl, 1% (v/v) TritonX-100, and Complete Mini Protease Inhibitor Cocktail (Roche, Indianapolis, IN)). Samples were incubated on ice for 30 minutes. Proteins concentrations were determined using a bicinchoninic acid protein assay kit (Pierce Biotechnology, Rockford, IL) and equal amounts of protein where separated into cytosol and cytoskeleton fractions by centrifugation at 100,000×*g* for 1 hour. Pellets (cytoskeleton fractions) were resuspended in 2X Laemmli buffer. Cytosol fractions (supernatant) were concentrated by precipitation with an equal volume of 20% (w/v) trichloroacetic acid for 30 min on ice, pelleted, washed with ice-cold acetone, dried, and resuspended in 2X Laemmli buffer. If necessary, residual trichloroacetic acid was neutralized with the addition of 1M Tris, pH 8. Cell protein fractions were then subjected to western blot analysis as described above. Densitometric quantitation of relative band intensity was performed using the NIH Image J program and normalized to cell number for total PKCβII levels.

### Western Blot Analysis

Cells were grown in 100 mm tissue culture dishes as described above, lysed with RIPA buffer [20 mM Tris-HCl pH 7.5, 1 mM EDTA, 150 mM NaCl, 1% Triton X-100, 0.5% Na-Deoxycholate, and 0.5% SDS, plus Complete Mini Protease Inhibitor Cocktail (Roche, Indianapolis, IN)], homogenized using a 22-gauge needle, and insoluble debris was cleared by centrifugation (15,000 *g*) for 20–30 minutes. Protein concentrations were determined using a bicinchoninic acid protein assay kit (Pierce Biotechnology, Rockford, IL). Proteins (10–20 µg/lane) were separated on 12–15% SDS polyacrylamide gels and transferred to a PVDF membrane (BioRad, Hercules, CA). PVDF membranes were blocked with 5% non-fat milk in wash buffer [10 mM Tris-HCl pH 7.5, 150 mM NaCl, 0.05% Tween-20]. Blots were immunostained with mouse monoclonal antibodies to total actin, vinculin, α-tubulin, β-tubulin, and γ-tubulin (Sigma, St. Louis, MO); Focal Adhesion Kinase (Upstate/Millipore, Billerica, Massachusetts); α–actinin (Abcam, Cambridge, MA); PKCβII and APC (Santa Cruz Biotechnology, Santa Cruz, CA). After incubation with horseradish peroxidase-conjugated secondary antibodies (Sigma, St. Louis, MO), SuperSignal West Femto Maximum Sensitivity Substrate (Thermo Fisher/Pierce Biotechnology, Inc., Rockford, IL) was used to visualize protein bands on the Chemidoc (Bio-Rad, Ventura, CA). Densitometric quantitation of relative band intensity was performed using the NIH Image J program and normalized to relative optical units of ribosomal protein L19 (RPL19) or γ–tubulin. Data is expressed as percent of controls and is the average of three biological replicates done in duplicate.

### Immunofluorescent staining

Cells were seeded on sterile glass coverslips as described previously [12] and fixed in either cold methanol for 4 minutes or 3% paraformaldehyde (PF) in 250 mM HEPES followed by a permeabilization step in 6% PF with 0.25% Triton X-100 in 250 mM HEPES for 10 minutes each at room temperature (RT). Cells were blocked with 2% chicken serum in PBS, incubated with primary antibodies (Phosphoserine, Pan-cytokeratin, Pan-cytokeratin FITC conjugate, FAK phospho-tyrosine 861 (Sigma, St. Louis, MO), Phospho-tyrosine (Zymed/Invitrogen, Carlsbad, CA), or listed above) for 20–60 minutes at RT, followed by three washes with PBS. Samples were incubated with appropriate secondary antibodies conjugated to Alexa Fluor^488^, Alexa Fluor^594^ (Molecular Probes, Eugene, OR) or TRITC (Sigma, St. Louis, MO) for 20 minutes at RT, followed by three washes with PBS. To stain actin, coverslips were incubated with Alexa Flouor^488^ conjugated phalloidin (Molecular Probes, Eugene, OR) for 20 minutes. Coverslips were mounted onto glass slides using Prolong Gold Antifade mounting medium with DAPI (Invitrogen, Carlsbad, CA).

Immunofluorescent micrographs were captured using a 60X objective on a Nikon 80*i* epifluorescence microscope equipped with UV, FITC and TRITC filters, and DS-Fi1 color and DS-U2 monochromatic cameras using NIS Elements BR 3.0 software (Nikon Instruments, Inc.) and processed with Adobe Photoshop®. To compare protein expression levels and subcellular localization, care was taken to ensure that micrographs were taken with the same exposure time. For confocal microscopy, immunofluorescently labeled cells were imaged with a Swept Field Confocal system (Prairie Technologies) on a Nikon Eclipse TE-2000U inverted microscope equipped with a 60×,1.4 NA Plan-Apochromatic phase–contrast objective lens and automated ProScan stage (Prior Scientific). The confocal head was equipped with filters for illumination at 488, 568, and 647 nm from a 400 mW argon laser and a 150 mW krypton laser. Digital images were acquired with an HQ2 CCD camera (Photometrics). Image acquisition, shutter, Z-axis position, laser lines, and confocal system were all controlled by NIS Elements AR software (Nikon). Z-series optical sections through each cell were obtained at 0.6 µm steps. Images were processed using Adobe Photoshop®.

### Quantitation of Filamentous Actin

Cells were seeded at 10,000 cells/well in a 24 well plate, and parallel plates were used to determine the mean cell number per well. Cells were fixed after 48 hours in 3% PF for 10 minutes followed by permeabilization in 6% PF containing 0.5% Triton X-100 for 10 minutes. Cells were quenched with 50 mM Glycine, and washed with PBS followed by a 60 min blocking step with 2% chicken serum for at least 60 minutes. F-actin was stained with Alexa Fluor^488^ conjugated phalloidin for 30 minutes, followed by extensive washing to remove unbound phalloiden. Alexa Fluor^488^ Phalloidin was subsequently solubilized with MeOH. Recovered fluorescence (Ex488/Em525) was determined using a safire2 microplate reader (Tecan, Durham, NC) with Magellan v6.3 for windows software (Tecan, Durham, NC). The amount of filamentous actin is expressed as the average relative fluorescence per cell ± the standard deviation calculated with a standard propagation of error equation σ^z^ =  square root [(σ^x^/average x)^2^ + (σ^y^/average y)^2^] x average z, where in this experiment z is the fluorescence/cell number, x fluorescence, y cell number, and σ the standard deviation [74].

## Supporting Information

Table S1 **Differentially expressed actin binding regulating genes in MOSE cell stages**
(DOC)

Table S2 **Differentially Expressed Microtubule and Microtubule Associated Genes in MOSE cell stages.**
(DOC)
