# Supplementary material for: Changes in Gene Expression and Cellular Architecture in an Ovarian Cancer Progression Model
Source: PLoS One. 2011 Mar 3;6(3):e17676. doi: 10.1371/journal.pone.0017676 (PMC3048403; doi:10.1371/journal.pone.0017676)
Supplement: Table S1 — Differentially expressed actin binding regulating genes in MOSE cell stages (DOC) [file pone.0017676.s001.doc]

**Supplemental Table S**1. Differentially expressed actin binding regulating genes in MOSE cell stages

| **Gene symbol** | **Gene Name** | **Accession Number** | **I/E** | **p-val** | **L/E** | **p-val** |
| --- | --- | --- | --- | --- | --- | --- |
| Actr3 | ARP3 actin-related protein 3 homolog (yeast) | NM_023735 | -1.4 | 0.0095 | -2.0 | 0.0102 |
| ***Akap12*** | ***A kinase (PRKA) anchor protein (gravin) 12*** | ***NM_031185*** | ***-9.9*** | ***0.0151*** | ***-11.8*** | ***0.0141*** |
| Akap2 | A kinase (PRKA) anchor protein 2 | NM_001035532 | -1.8 | 0.0000 | -2.3 | 0.0001 |
| Anln | anillin, actin binding protein | NM_028390 | -1.5 | 0.0408 | -2.5 | 0.0144 |
| Arhgap24 | Rho GTPase activating protein 24 | NM_029270 | -8.5 | 0.0096 | -39.4 | 0.0059 |
| Arhgap6 | Rho GTPase activating protein 6 | NM_009707 | 2.5 | 0.1591 | 12.8 | 0.0033 |
| Arpc5l | actin related protein 2/3 complex, subunit 5-like | NM_028809 | 1.7 | 0.0110 | 2.5 | 0.0150 |
| Cap1^+^ | CAP, adenylate cyclase-associated protein 1 | NM_007598 | -2.5 | 0.0014 | -2.1 | 0.0260 |
| Cdc42ep2 | CDC42 effector protein (Rho GTPase binding) 2 | NM_026772 | 1.5 | 0.2568 | -3.1 | 0.0029 |
| Cdc42ep3 | CDC42 effector protein (Rho GTPase binding) 3 | NM_026514 | -1.8 | 0.0276 | -3.1 | 0.0063 |
| Cdc42ep5 | CDC42 effector protein (Rho GTPase binding) 5 | NM_021454 | 1.1 | 0.7844 | -3.6 | 0.0013 |
| Cnn2 | calponin 2 | NM_007725 | -2.3 | 0.0765 | -7.5 | 0.0195 |
| Coro1b | coronin, actin binding protein 1B | NM_011778 | 2.0 | 0.0011 | 2.4 | 0.0424 |
| Coro1c | coronin, actin binding protein 1C | NM_011779 | -1.4 | 0.0468 | -2.5 | 0.0196 |
| Csrp1 | cysteine and glycine-rich protein 1 | NM_007791 | -2.4 | 0.0717 | -3.3 | 0.0462 |
| Dbn1^*^ | drebrin 1 | NM_019813 | -2.1 | 0.0034 | -2.1 | 0.0021 |
| ***Diap3*** | ***diaphanous homolog 3 (Drosophila)*** | ***NM_019670*** | ***-2.1*** | ***0.0016*** | ***-4.2*** | ***0.0045*** |
| Ehd2 | EH-domain containing 2 | NM_153068 | -1.2 | 0.3490 | -2.2 | 0.0401 |
| Enc1 | ectodermal-neural cortex 1 | NM_007930 | 1.1 | 0.1596 | 2.8 | 0.0042 |
| Epb4.1l1 | erythrocyte protein band 4.1-like 1 | NM_013510 | 3.1 | 0.0171 | 3.6 | 0.0272 |
| Epb4.1l4a | erythrocyte protein band 4.1-like 4a | NM_013512 | 4.1 | 0.1356 | 4.3 | 0.0001 |
| Evl | Ena-vasodilator stimulated phosphoprotein | NM_007965 | -1.3 | 0.1199 | -2.6 | 0.0043 |
| Fmn1^*^ | formin 1 | NM_010230 | -2.5 | 0.0280 | -2.4 | 0.0478 |
| Fyn | Fyn proto-oncogene | NM_001122893 | 1.4 | 0.1021 | 2.7 | 0.0362 |
| ***Flnb^+^*** | ***filamin, beta*** | ***NM_134080*** | ***-5.3*** | ***0.0283*** | ***-4.0*** | ***0.0385*** |
| Fscn1 | fascin homolog 1, actin bundling protein | NM_007984 | -1.5 | 0.0487 | -4.1 | 0.002 |
| ***Gsn*** | ***Gelsolin*** | ***NM_146120*** | ***1.2*** | ***0.0496*** | ***2.4*** | ***0.0284*** |
| ***IQGAP2*** | ***IQ motif containing GTPase actinvating protein 2*** | ***NM_027711*** | ***3.6*** | ***0.4092*** | ***14.0*** | ***0.0185*** |
| *IQGAP3* | *IQ motif containing GTPase actinvating protein 3* | *NM_178229* | *-2.1* | *0.0140* | *-2.1* | *0.0987* |
| Ivns1abp | influenza virus NS1A binding protein^+^ | NM_001039511 | -2.6 | 0.0134 | -2.1 | 0.0233 |
| Kalrn | kalirin, RhoGEF kinase | XM_001481029 | -1.7 | 0.0267 | -2.4 | 0.0039 |
| Kras | Kirsten rat sarcoma viral oncogene homolog | NM_021284 | -1.1 | 0.1181 | -2.8 | 0.0008 |
| Lmo7^+^ | LIM domain only 7 | NM_201529 | -3.7 | 0.0008 | -2.7 | 0.0104 |
| Map2k1 | mitogen activated protein kinase kinase 1 | NM_008927 | 1.4 | 0.0781 | 2.2 | 0.0236 |
| Map2k5 | mitogen activated protein kinase kinase 5 | NM_011840 | 1.3 | 0.1457 | 2.0 | 0.0045 |
| Map3k1 | mitogen activated protein kinase kinase kinase 1 | NM_011945 | 1.1 | 0.5470 | 2.1 | 0.0028 |
| Marcks | Myristoylated alanine-rich kinaseC substrat | NM_008538 | -1.6 | 0.0059 | -2.1 | 0.0059 |
| ***Msn*** | ***Moesin*** | ***NM_010833*** | ***-1.8*** | ***0.0012*** | ***-2.4*** | ***0.0007*** |
| Mtss1 | metastasis suppressor 1 | NM_144800 | 1.7 | 0.2052 | -2.2 | 0.0114 |
| Myh10 | myosin, heavy polypeptide 10, non-muscle | NM_175260 | -2.4 | 0.0770 | -4.0 | 0.0264 |
| Myh9 | myosin, heavy polypeptide 9, non-muscle | NM_022410 | -2.3 | 0.0567 | -2.3 | 0.0558 |
| Mylip | myosin regulatory light chain interacting protein | NM_153789 | -1.1 | 0.5508 | -2.2 | 0.0042 |
| Myo18a | myosin XVIIIa | NM_011586 | 1.2 | 0.0760 | 2.9 | 0.0281 |
| Myo1c | myosin IC | NM_001080775 | -2.6 | 0.0165 | -3.2 | 0.0135 |
| Myo1d | myosin ID | NM_177390 | 1.1 | 0.4898 | 2.1 | 0.002 |
| Palld | palladin, cytoskeletal associated protein | NM_001081390 | -1.9 | 0.0531 | -3.1 | 0.0145 |
| Pdlim1 | PDZ and LIM domain 1 (elfin) | NM_016861 | 1.0 | 0.8789 | -3.0 | 0.0012 |
| Pdlim5 | PDZ and LIM domain 5 | NM_019808 | -2.4 | 0.0045 | -3.0 | 0.0025 |
| Pdlim7 | PDZ and LIM domain 7 | NM_001114088 | -2.6 | 0.0168 | -4.1 | 0.0051 |
| Plcb1 | phospholipase C, beta 1 | NM_019677 | 1.5 | 0.2930 | 4.4 | 0.0015 |
| Plcb4 | phospholipase C, beta 4 | NM_013829 | 1.7 | 0.1110 | 3.2 | 0.0165 |
| Plec1 | plectin 1 | NM_011117 | 1.7 | 0.2388 | 3.2 | 0.0111 |
| Pls3 | plastin 3 (T-isoform) | NM_145629 | -2.0 | 0.0016 | -3.1 | 0.0002 |
| Ptpn13 | tyrosine phosphatase, non-receptor type 13 | NM_011204 | -1.9 | 0.0118 | 2.8 | 0.0004 |
| Rai14 | retinoic acid induced 14 | NM_030690 | 2.0 | 0.0999 | 2.3 | 0.0066 |
| Rassf5 | Ras association (RalGDS/AF-6) domain family5^+^ | NM_018750 | -20.0 | 0.0177 | -2.1 | 0.0083 |
| Rhoj | ras homolog gene family, member J | NM_023275 | -2.7 | 0.0256 | -7.5 | 0.0077 |
| Rhou | ras homolog gene family, member U | NM_133955 | 1.2 | 0.3921 | 2.3 | 0.0257 |
| Rps6ka1 | ribosomal protein S6 kinase polypeptide 1 | NM_009097 | 3.8 | 0.0006 | 2.8 | 0.0076 |
| Sdc1 | syndecan 1 | NM_011519 | 2.1 | 0.1855 | 2.5 | 0.0391 |
| Sept11 | septin 11 | NM_001009818 | -1.5 | 0.0405 | -2.2 | 0.0181 |
| Sgce | sarcoglycan, epsilon | NM_001130188 | -1.2 | 0.7446 | -41.8 | 0.0016 |
| Sh3kbp1 | SH3-domain kinase binding protein 1 | NM_001135727 | -1.6 | 0.0591 | -6.9 | 0.0007 |
| Sorbs1 | sorbin and SH3 domain containing 1^+^ | NM_009166 | -28.3 | 0.0496 | -15.4 | 0.0545 |
| Spire1 | spire homolog 1 (Drosophila) | NM_176832 | -1.9 | 0.0270 | -2.1 | 0.0189 |
| Spry2 | sprouty homolog 2 (Drosophila) | NM_011897 | -1.2 | 0.5382 | 2.7 | 0.0159 |
| Syne2 | synaptic nuclear envelope 2 | NM_001005510 | -1.5 | 0.1919 | 2.9 | 0.0289 |
| ***Tpm2*** | ***Tropomyosin 2, beta*** | ***NM_009416*** | ***-1.9*** | ***0.2948*** | ***-42.1*** | ***0.0191*** |
| Tpm3 | Tropomyosin 3, gamma | NM_022314 | -1.4 | 0.1108 | -2.3 | 0.0069 |
| Tpm4 | tropomyosin 4 | NM_001001491 | -1.4 | 0.1012 | -2.4 | 0.0165 |
| ***Wasl*** | ***Wiskott-Aldrich syndrome-like (human)*** | ***NM_028459*** | ***1.0*** | ***0.7863*** | ***2.1*** | ***0.0008*** |
| Wdr1 | WD repeat domain 1 | NM_011715 | -1.1 | 0.6296 | -2.3 | 0.0126 |

List of genes differentially regulated which are structural or regulatory proteins of the actin cytoskeleton. Fold differences ≥ 2 with p< 0.05 are considered significant. Genes in italics were analyzed by RT-PCR and those in bold were validated to change significantly (p<0.05) between MOSE-E and MOSE-L cells and those not in bold were validated to change significantly (p<0.05) between MOSE-E and MOSE-I cells. Genes that have the greatest significant changes in MOSE-I cells are indicated by a plus (+). Genes indicated by an asterisk (*) have MOSE-I/MOSE-E ratios that are within less than 0.4 fold of MOSE-L/MOSE-E ratios.
