# Supplementary material for: Changes in Gene Expression and Cellular Architecture in an Ovarian Cancer Progression Model
Source: PLoS One. 2011 Mar 3;6(3):e17676. doi: 10.1371/journal.pone.0017676 (PMC3048403; doi:10.1371/journal.pone.0017676)
Supplement: Table S2 — Differentially Expressed Microtubule and Microtubule Associated Genes in MOSE cell stages. (DOC) [file pone.0017676.s002.doc]

**Supplemental**

**Table S2. Differentially Expressed Microtubule and Microtubule Associated Genes in MOSE cell stages.**

| **Gene Symbol** | **Gene Name** | **Accession Number** | **I/E** | **p-val** | **L/E** | **p-val** |
| --- | --- | --- | --- | --- | --- | --- |
| **Microtubule** |  |  |  |  |  |  |
| Tuba4a | tubulin, alpha 4A | NM_009447 | 1.5 | 0.0301 | -2.2 | 0.0146 |
| Tubb2a | tubulin, beta 2a | NM_009450 | -1.2 | 0.4725 | -3.5 | 0.0007 |
| Tubb2b | tubulin, beta 2b | NM_023716 | -2.8 | 0.0200 | -3.3 | 0.0144 |
| Tubb2c | tubulin, beta 2c | NM_146116 | 1.2 | 0.2601 | -2.1 | 0.0261 |
| ***Tubb3*** | ***Tubulin, beta 3*** | ***NM_023279*** | ***1.5*** | ***0.1902*** | ***-3.0*** | ***0.0362*** |
| Tubb6 | tubulin, beta 6 | NM_026473 | -1.2 | 0.0437 | -4.7 | 0.0013 |
| **Microtubule Binding and Regulation** | |  |  |  |  |  |
| Aspm* | asp (abnormal spindle)-like | NM_009791 | -1.7 | 0.0467 | -3.0 | 0.0300 |
| Bicd2 | bicaudal D homolog 2 (Drosophila) | NM_001039179 | -1.9 | 0.0337 | -2.1 | 0.0282 |
| Cdc6 | cell division cycle 6 homolog (S. cerevisiae) | NM_011799 | 1.4 | 0.0953 | -2.6 | 0.0248 |
| Cdgap | CDC42 GTPase-activating protein | NM_020260 | -1.4 | 0.0333 | -2.5 | 0.0021 |
| Cenpe* | centromere protein E | NM_173762 | -2.2 | 0.0246 | -3.7 | 0.0171 |
| Ckap2* | cytoskeleton associated protein 2 | NM_001004140 | 1.1 | 0.3932 | -2.2 | 0.0324 |
| Ckap2l | cytoskeleton associated protein 2-like | NM_181589 | 1.0 | 0.2245 | -2.8 | 0.0050 |
| Ckap4 | cytoskeleton-associated protein 4 | NM_175451 | -1.7 | 0.0290 | -3.0 | 0.0077 |
| Dnm2 | dynamin 2 | NM_001039520 | 1.7 | 0.2847 | 4.4 | 0.0049 |
| Dync1i1 | dynein cytoplasmic 1 intermediate chain 1 | NM_010063 | 1.4 | 0.4035 | 6.1 | 0.0450 |
| Fam110c | family with sequence similarity 110, member C | NM_027828 | -1.9 | 0.0559 | -2.1 | 0.0346 |
| Incenp* | inner centromere protein | NM_016692 | 1.1 | 0.1875 | -2.1 | 0.0379 |
| Jub* | Ajuba | NM_010590 | -3.6 | 0.0390 | -3.2 | 0.04509 |
| Kif1b | kinesin family member 1B | NM_008441 | -1.9 | 0.0304 | -2.3 | 0.0195 |
| ***Kif18a**** | ***kinesin family member 18A*** | ***NM_139303*** | ***-1.2*** | ***0.2028*** | ***-2.8*** | ***0.0062*** |
| ***Kif20a**** | ***kinesin family member 20A*** | ***NM_009004*** | ***1.2*** | ***0.1513*** | ***-2.7*** | ***0.0331*** |
| Kif21a | kinesin family member 21A | NM_016705 | 1.5 | 0.2060 | 2.4 | 0.0334 |
| *Kif22** | *Kinesin family member 22* | *NM_145588* | *1.0* | *0.2291* | *-2.7* | *0.0178* |
| ***Kif23**** | ***kinesin family member 23*** | ***NM_024245*** | ***-1.2*** | ***0.0970*** | ***-3.4*** | ***0.0082*** |
| Kif26b | kinesin family member 26B | NM_001161665 | 1.0 | 0.9068 | -3.9 | 0.0102 |
| Kif2c | kinesin family member 2C | NM_134471 | -1.2 | 0.0918 | -2.9 | 0.0211 |
| Kif4* | kinesin family member 4 | NM_008446 | -1.4 | 0.0966 | -2.1 | 0.0306 |
| Klc1 | Kinesin light chain 1 | NM_008450 | -1.4 | 0.0455 | -2.6 | 0.0009 |
| Klc4 | Kinesin light chain 4 | NM_029091 | 1.3 | 0.3611 | 3.1 | 0.0015 |
| Lats2* | large tumor suppressor 2 | NM_015771 | -3.4 | 0.0035 | -2.7 | 0.0058 |
| Map1lc3b | microtubule-associate1 light chain 3 beta | NM_026160 | 1.7 | 0.0930 | 2.3 | 0.0466 |
| Mtap6 | microtubule-associated protein 6 | NM_010837 | -3.0 | 0.0012 | -15.7 | 0.0004 |
| Ndn | Necdin | NM_010882 | -3.7 | 0.0124 | -26.8 | 0.0017 |
| Ninl^+^ | ninein-like | NM_207204 | -2.6 | 0.0068 | -2.2 | 0.0029 |
| Nudc | Nuclear distribution gene C homolog | NM_010948 | -1.3 | 0.1131 | -2.1 | 0.0109 |
| Pea15a | phosphoprotein enriched in astrocytes 15A | NM_011063 | -1.3 | 0.2698 | -2.9 | 0.0179 |
| Prc1* | protein regulator of cytokinesis 1 | NM_145150 | -1.4 | 0.0245 | -2.6 | 0.0158 |
| Shroom3 | Shroom family member 3 | NM_015756 | -1.2 | 0.3917 | 2.8 | 0.0115 |
| Spag5 | sperm associated antigen 5 | NM_017407 | -1.3 | 0.2226 | -2.2 | 0.0313 |
| Tbcel | Tubulin folding cofactor E-like | NM_173038 | 1.2 | 0.1501 | 3.5 | 0.0292 |
| Tpx2 | TPX2, microtubule-associated protein homolog | NM_001141977 | -1.2 | 0.0319 | -2.2 | 0.0194 |
| Vav2 | vav 2 guanine nucleotide exchange factor | NM_009500 | -2.1 | 0.0226 | -4.0 | 0.0062 |

List of genes differentially regulated which are structural or regulatory proteins of the microtubule network. Fold differences ≥ 2 with p< 0.05 are considered significant. Genes in italics were analyzed by RT-PCR and those in bold were validated to change significantly (p<0.05). Genes indicated by a plus (+) have MOSE-I/MOSE-E ratios that are within less than 0.4 fold of MOSE-L/MOSE-E ratios. An asterisk indicates genes that have products involved in chromosome congression, segregation or cytokinesis.
